# Association of bone mineral density with biochemical markers of bone turnover in hemodialysis children

**DOI:** 10.15171/jrip.2016.37

**Published:** 2016-07-27

**Authors:** Niloofar Hajizadeh, Mehryar Mehrkash, Daryoosh Fahimi, Mostafa Qorbani, Nina Shafa

**Affiliations:** ^1^Department of Pediatric Nephrology, Children’s Medical Center, Tehran University of Medical Sciences, Tehran, Iran; ^2^Department of Pediatric Nephrology, Isfahan University of Medical Sciences, Isfahan, Iran; ^3^Department of Pediatric Nephrology, Bahrami Children Hospital, Tehran University of Medical Sciences, Tehran, Iran; ^4^Department of Community Medicine, School of Medicine, Alborz University of Medical Sciences, Karaj, Iran; ^5^Iranian Resaerch Center on Ageing, University of Social Walfare and RehabilitationSciences,Tehran, Iran

**Keywords:** Chronic kidney disease, Bone mineral density, Hemodialysis, Children

## Abstract

**Introduction:** Although some descriptive and cross-sectional studies have been reported about bone mass in chronic kidney disease (CKD) children, only a few studies investigated markers of bone turnover and the bone mass measurements.

**Objectives:** The aim of this study was to evaluate the association between bone mineral density (BMD) and biochemical markers of bone turnover in hemodialysis (HD) children.

**Patients and Methods:** The children who had received dialysis for at least the preceding 6‐month were included. BMD was measured for total body, the lumbar spine and the femoral neck and the blood samples were tested to assess biochemical bone turnover markers.

**Results:** The study group was comprised of 27 patients with CKD, 9 males (33%) and 18 females (67%) with the mean±SD age of the subjects 14.9±4.5 years. Positive significant correlations of parathyroid hormone (PTH) with total body bone densitometry Z-score, lumbar spine and femoral neck Z-score(r=0.43, *P*=0.06; r=0.41, *P*=0.08 and r=0.45, *P*=0.05, respectively) was noted. In addition, positive significant correlations calcium and total body, lumbar spine and femoral neck Z-score (r=0.52, *P*=0.02; r=0.28, *P*=0.23 and r=0.36, *P*=0.12, respectively) was seen. Interestingly, a positive significant correlation between alkaline phosphatase (ALP) and lumbar spine Z-score was found (r=0.46, *P*=0.04), while the correlation of this parameter with total body and femoral neck Z-score was not significant (*P*>0.05).

**Conclusion:** In our study, majority of patients with CKD had low level of BMD. In addition, lower levels of calcium (Ca), phosphorus (P), PTH and 25 (OH) vitamins D in patients with abnormal BMD Z-scores were detected.

**Implication for health policy/practice/research/medical education:**

In a study on 27 patients with chronic kidney disease (CKD), with the age of 14.9 ± 4.5 years, we found, positive correlations of parathyroid hormone (PTH) with total body, lumbar spine and femoral neck Z-score. In addition, a positive correlation between calcium and total body, lumbar spine and femoral neck Z-score was found. Interestingly, positive correlations between alkaline phosphatase (ALP) and lumbar spine Z-score was found too, while the correlation of this parameter with total body and femoral neck Z-score was not significant. In our study a majority of patients with CKD had low level of bone mineral density (BMD). In addition, lower levels of calcium (Ca), phosphorus (P) PTH, 25 (OH) vitamins D were found in patient with abnormal BMD Z-scores.

## Introduction

The syndrome known as chronic kidney disease–mineral and bone disorder (CKD-MBD) is composed of clinical, biochemical and radiological abnormalities associated with CKD, that is manifested by either one or a combination of the following factors: abnormalities of calcium, phosphorus, parathyroid hormone (PTH), or vitamin D metabolism, abnormalities in bone histology, linear growth, or strength, and vascular or other soft tissue calcification (1).

Although some descriptive and cross-sectional studies have been reported about bone mass in CKD children, only a few studies include have been reported markers of bone turnover and the bone mass measurements (2-6). On the other hand, however, there are the numerous investigations on mineral and bone metabolism in hemodialysis (HD) patients aged between 50 and 80 years. A lack of studies in young HD patients are existed (7).

Bone mineral density (BMD) and bone turnover are the most important factors in the classification of patients with CKD (8). It has been reported controlling of biochemical parameters of CKD-MBD (e.g., serum calcium, phosphorus, and PTH levels) is so crucial in these patients (9). Since signaling mechanisms between bone, kidney, and parathyroid glands, alterations in kidney function can prompt to changes in serum biochemical values (1).

## Objectives

The aim of this study was to evaluate the relationship between BMD and biochemical markers of bone turnover in HD children.

## Patients and Methods

### Subjects

Patients were recruited from the Children Medical Center, an Iranian referral pediatric Hospital, and Bahrami hospital, Tehran, Iran. The children who had received dialysis for at least the preceding 6‐month were included.

BMD was measured for total body, the lumbar spine and the femoral neck with the use of the discovery osteodensitometer (S/N 84289) and the Z-scores (age and gender specific) were calculated.

Blood samples tested to assess biochemical bone turnover markers were collected before the HD session in the morning. For each patient, the mean of the results of each parameter during 3 months were calculated and reported. Serum levels of calcium (Ca), phosphorus (P), zinc, creatinine, hemoglobin, albumin, alkaline phosphatase (ALP), total cholesterol, triglyceride, uric acid, folic acid, ferritin, total iron binding capacity (TIBC), vitamin B12, 25 (OH) vitamin D were measured using standard methods (IDS ELISA kit). Plasma bicarbonate (HCO3) and blood pH were assessed in heparinized arterial blood with the use of the radiometer (ABL 5 blood gas analyzer, Denmark). Serum whole PTH levels measured with an electrochemiluminescence immunoassay (Elecsys, Roche, Germany). Plasma albumin was measured using colorimetry and defined as low at a value <3.5 g/dL. Urea clearance time × dialysis time/volume (Kt/V) values of all patients were measured and calculated with the use of the Daugirdas formula (10).

Conservative treatment was given when indicated with sodium bicarbonate, calcium carbonate and alphacalcidol but no patients received aluminum containing phosphate binders. In addition, antihypertensive treatment was given when indicated.

### Ethical issues

The research followed the tenets of the Declaration of Helsinki; informed consent was obtained; and the research was approved by the ethical committee of Tehran University of Medical Sciences.

### Statistical analysis

The continuous variables are presented as mean± SD and categorical variable as number and percentage. The association between categorical variable was assessed using chi-square tests. Comparison of biochemical parameters mean with Z-scores status was assessed using *t* test. Correlations of BMD and other parameters were assessed by Spearman’s rank correlation coefficient. Analysis was performed in SPSS version 16 software and *P* value less than 0.05 was considered as statistically significant.

## Results

The study group was comprised of 27 patients with CKD, consisting 9 males (33%) and 18 females (67%) with the age of 14.9 ± 4.5 years.

### Results of the biochemical markers

Twenty-three of 27 patients (85%) had abnormal PTH levels (mean 214.4). Eighteen patients (67%) had normal phosphorus (3.6-5.8) and nearly half of the patients (44%) had normal 1,25-dihydroxyvitamin D levels>30. Most patients (89%) had abnormal HCO3 levels <22 (mean 16.3), but normal zinc (50<zn<150) (96%), cholesterol <200 (89%), triglyceride <175 (74%), iron 37-145 (78%) and ferritin 10-400 (70%) values. Normal ALP level < 300 was found in 16 patients (59%).

Fifty-six percent of patients (n=15) had adequate levels of calcium (mean 8.9±0.83) serum. In 67% (n=18) of patients, phosphatemia was adequate (mean 5.7±1.7 mg/dL) with a minimum of 3.5 mg/dL and a maximum of 12 mg/dL. Mean ALP was 385±350, with a minimum of 94 and a maximum of 1450 IU/L. The mean level of PTH was 219±214 ρg/mL, (range from 215 to 1011 ρg/mL). Only 15% of patients (n = 4) had levels suggested by the KDOQI guidelines (200-300 ρg/mL), 55% (n=15) had values below those suggested by the guidelines and 30% (n = 8) above 300 ρg/mL. According to the values suggested by the KDOQI guidelines, 44% (n=12) had adequate levels of 25 (OH) D3, and 24 patients (89%) have abnormal HCO3 level. While it is recommended in HD patients that HCO3 be kept above 22 but in this study there was no association between CKD-MBD and HCO3.

The dialysis adequacy was very good (85%); however, there was no significant association between adequacy of dialysis and CKD-MBD osteoporotic or osteopenia patients.

### Bone mineral density

The mean (SD) of the BMD (Z-score) of the total body was -2.4 (SD=1.5), of the lumbar spine was -2.3 (SD=2.3) and of the femoral neck was -2.58 (SD=1.5).

For male patients the mean Z-score of the BMD of the total body was -3 (SD=0.64), lumbar spine was -2.9 (SD=1.5) and the femoral neck was -2.8 (SD=0.87). In female patients the Z-score of the BMD of the total body was -2.2 (SD=1.6), the lumbar spine was -2 (SD=2.5) and the femoral neck was -2.5 (SD=1.6).

A total body, lumbar spine and femoral neck Z-score of between -1 and -2.5 SD, consistent with osteopenia, was found in 8 (40%), 6 (30%) and 6 (30%) of all 20 patients measured, respectively; 9 patients (45%) had a total and lumbar spine Z-score of less than -2.5 SD, while 12 (60%) had a femoral neck Z-score of less than -2.5 SD that were considered as osteoporosis (Table 1).

**Table 1:** The frequency of BMD based on different Z-scores in hemodialysis children

**Z score**	**Total body**		**Lumbar spine**		**Femoral neck**	
	**n**	**%**	**n**	**%**	**n**	**%**
Between -1 and 1 SD	3	15	5	25	2	10
Between -2.5 and -1 SD	8	40	6	30	6	30
Less than -2.5 SD	9	45	9	45	12	60

Positive correlations between PTH and total body, lumbar spine and femoral neck Z-score (*r*=0.43, *P*=0.06; *r*=0.41, *P*=0.08 and *r*=0.45, *P*=0.05, respectively) were noted. In addition, a positive correlation was found between calcium and total body, lumbar spine and femoral neck Z-score (*r*=0.52, *P*=0.02; *r*=0.28, *P*=0.23; *r*=0.36, *P*=0.12, respectively) (Table 2).

**Table 2:** Correlation of BMD (Z-scores) and biochemical markers of bone turnover in hemodialysis children in Tehran, Iran

	**Lumbar spine Z-score**		**Femoral neck Z-score**		**Total Z-score**	
	**Correlation Coefficient (r)**	***P*** ** value**	**Correlation Coefficient (r)**	***P*** ** value**	**Correlation Coefficient (r)**	***P*** ** value**
Ca (mg/dL)	0.28	0.23	0.36	0.12	0.52	0.02
PTH (pg/mL)	0.41	0.08	0.45	0.05	0.43	0.06
Folic acid (ng/mL)	0	1	0	0.7	0.1	0.8
Vitamin B12 (pg/mL)	-0.1	0.76	0.04	0.9	0.19	0.55
Vitamin D (ng/mL)	-0.1	0.53	0.09	0.7	0	0.87
Zinc (µg/dL)	0.2	0.5	0	0.9	0.1	0.8
PH	-0.2	0.4	-0.2	0.4	-0.1	0.6
HCO3 (mmol/l)	-0.08	0.74	-0.12	0.61	0.04	0.86
Hb (g/dL)	-0.33	0.15	-0.07	0.77	-0.17	0.46
Creatinine (mg/dL)	0.02	0.95	0.19	0.43	0.2	0.41
P (mg/dL)	0.38	0.09	0.24	0.3	0.36	0.12
ALP (U/L)	0.46	0.04	0.28	0.23	0.19	0.43
Cholesterol (mg/dL)	0.3	0.19	0.1	0.67	0.07	0.76
Triglyceride (mg/dL)	0.2	0.4	0	0.5	0	0.6
Uric acid (mg/mL)	0.21	0.38	0.26	0.28	0.22	0.36
Iron (µg/dL)	0.3	0.3	0	0.9	0.1	0.7
TIBC (µg/dL)	0	0.92	0.16	0.51	0.08	0.75
Ferritin (ng/mL)	0.2	0.4	0	0.9	0.2	0.5
Alb (g/dL)	0.23	0.34	0.22	0.36	0.34	0.14
KT/V	0	0.1	-1	0	0	0.1

Interestingly, positive correlation of ALP and lumbar spine neck Z-score was found (*r*=0.46, *P*=0.04), while the correlation of this parameter with total body and femoral neck Z-score was not significant (*r*=0.19, *P*=0.43; *r*=0.28, *P*=0.23, respectively) (Table 2). No statistically significant relationship between BMD (Z-score) and other parameters was observed.

Lower phosphorus and vitamin B12 were found in patients with abnormal BMD Z- scores. However there was not any statistically significant relationship (Table 2). In addition, the lower level of creatinine and ferritin was found in patients with abnormal total body Z-score (7±1.6, *P*=0.039; and 260±244, *P*=0.028, respectively). Higher levels of HCO3 were found in children with abnormal femoral Z-scores (*P*= 0.05; 16.4±4.2). On the other hand there was no significant change of HCO3 sighted in patients having abnormal lumbar spine, or total body Z-scores (Table 3).

**Table 3:** Biochemical markers of bone and mineral metabolism in children with CKD based on different Z-scores

	**Z-femor**			**Z-lumbar**			**Z-total**		
	**Normal**	**Abnormal**	***P*** ** value**	**Normal**	**Abnormal**	***P*** ** value**	**Normal**	**Abnormal**	***P*** ** value**
Hco3 (mmol/l)	10 ± 5	16.7 ± 4.2	0.05	14.8 ± 5.5	16.4 ± 4.4		14.2 ± 7.5	16.3 ± 4.2	
Hb (g/dL)	8.1 ± 0.42	10 ± 1.5		9.1 ± 1.3	10.1 ± 1.5		8.9 ± 1.5	10 ± 1.5	
Creatinin (mg/dL)	8.6 ± 0.63	7.3 ± 2		7.7 ± 1	7.3 ± 2.3		9.6 ± 2.9	7 ± 1.6	0.039
Ca (mg/dL)	9.9 ± 0.56	8.7 ± 0.77	0.06	9.46 ± 0.8	8.7 ± 0.7	0.06	9.8 ± 0.4	8.7 ± 0.75	0.024
P (mg/dL)	8.6 ± 4	5.2 ± 0.9	0.003	7.1 ± 2.5	5 ± 0.9	0.01	7.9 ± 3.2	5.1 ± 0.9	0.005
ALP (U/L)	532 ± 406	412 ± 392		515 ± 321	393 ± 408		257 ± 120	453 ± 409	
Cholesterol (mg/dL)	173 ± 7	138 ± 38		165 ± 42	133 ± 34		168 ± 48	137 ± 35	
Triglyceride (mg/dL)	183 ± 52	117 ± 56		129 ± 62	121 ± 59		118 ± 88	124 ± 55	
Uric acid (mg/mL)	7.8 ± 1.1	9.1 ± 9		6.9 ± 2	9.6 ± 10.2		8.3 ± 1.2	9 ± 9.7	
Iron (µg/dL)	82 ± 51	183 ± 336		242 ± 342	150 ± 321		108 ± 40	185 ± 347	
TIBC (µg/dL)	202 ± 24	203 ± 63		188 ± 80	208 ± 53		247 ± 52	196 ± 59	
Ferritin (ng/mL)	589 ± 608	287 ± 243		491 ± 349	260 ± 247		642 ± 328	260 ± 244	0.028
Alb (g/dL)	4.1 ± 0.84	3.7 ± 0.6		4.1 ± 0.53	3.7 ± 0.6		4.1 ± 0.5	3.7 ± 0.6	
PTH (pg/mL)	711 ± 424	201 ± 166	0.002	475 ± 354	177 ± 142	0.013	320 ± 243	240 ± 248	
Folicacid (ng/mL)	11.5 ± 10	7.3 ± 3.3		10.3 ± 7.7	7.2 ± 3.5		13.5 ± 7.8	7 ± 3.4	0.067
Vitamin B12 (pg/mL)	901 ± 934	345 ± 160	0.07	711 ± 802	347 ± 170	0.19	981 ± 921	330 ± 170	0.03
Vitamin D(ng/mL)	23 ± 21	25.8 ± 11		23.3 ± 13.3	26.3 ± 11.8		18.8 ± 13	27 ± 11.7	
Zinc (µg/dL)	95 ± 36	81 ± 31		84.6 ± 25	82 ± 33.5		95 ± 28	80 ± 32	

## Discussion

Biochemical markers of bone turnover could be considered as early indicators of diagnostic and/or monitoring of CKD-MBD (6). The prevalence and causes of CKD might vary from one geographic area to another due to genetic and environmental factors (11).

According to Gheissari et al report, the annual incidences of CKD (mostly advanced stages) were 5.52 per million population (PMP) and 16.8 per million children population over 11 years in a tertiary care center in Isfahan province of Iran, respectively (12).

Patients with renal failure, especially those who are undergoing HD, are at high risk for low BMD and fracture because of associated risk factors such as a sedentary lifestyle; the use of drugs such as heparin, corticosteroids, and immunosuppressants; acidosis; and uremic toxins (13,14). In our study, the majority of patients had low level of BMD (90%, 85% and 75% according to femoral neck Z-score, total body Z-score and lumbar spine Z score, respectively). In Swolin‐Eide et al study, most markers of bone turnover were within the reference intervals and most of the children with CKD had an adequate BMD; however, a normal BMD did not exclude MBD (6).

In our study, nine patients (33%) had abnormal phosphate level. Although in the early stages of CKD, elevated amounts of circulating PTH may result in normal or low serum phosphate levels, in advanced stages, decreased glomerular filtration rate limits phosphorus excretion (1). It is well known that serum ALP can be used as a biochemical marker of high-turnover bone disease (15).

Serum ALP values are fair markers of osteoblastic activity in children with CKD. Osteoblasts normally express large amounts of the bone isoenzyme of ALP, and elevated serum levels correlate with increased bone formation, high levels of serum PTH, and growth hormone therapy (16). ALP is present in the liver and elevated serum levels of total ALP may not always indicate increased bone turnover (1). Therefore, we considered the mean serum level of this parameter during 3 months and found a positive correlations between the mean level of ALP and lumbar spine Z-score (*r*=0.46, *P*=0.04).

In addition, serum PTH levels are not reliable indicators of bone turnover in patients which are on long-term dialysis. This may be due to the daily variations of serum PTH levels (16). We considered the mean level of each parameter during 3 months. In our study, lower Ca, P, PTH, 25 (OH) D levels were found in patients with abnormal BMD Z-scores. In Denburg et al study, lower calcium, 25(OH)D and higher PTH and 1,25(OH)₂D were independently associated with lower cortical volumetric BMD Z-scores at baseline (17).

In our study, the mean level of PTH was positively correlated with Z-scores (*r*=0.43, *P*=0.06 for total body Z-score; *r*=0.41, *P*=0.08 for lumbar spine Z-score; and *r*=0.45, *P*=0.05 for femoral neck Z-score). In Wetzsteon et al study, among CKD participants, PTH levels were positively associated with trabecular BMD Z-score (*P*<0.01) (18). In Sit et al study, no statistically significant relationship was observed between BMD and biochemical markers of bone turnover (19), while in the study of Wetzsteon et al, higher levels of PTH and biomarkers of bone formation (bone-specific ALP) were associated with lower cortical BMD Z-scores (*P*≤0.02) (18). In Swolin-Eide et al study, 11 CKD patients (73%) had increased PTH levels at baseline and most children had normal levels vitamin D and it was not found any correlation between Z-scores and the severity of CKD (6).

Although 25(OH) vitamin D deficiency is common in children with CKD (1), in this study only 6 patients (22%) had vitamin D deficiency. Levels of both serum 25(OH)D and 1,25 dihydroxyvitamin D (1,25[OH]2D), the most active form of vitamin D, are known to decrease with decreasing glomerular filtration rate (GFR) in patients with CKD (20). Serum 25 (OH)_2_ D3 levels were lower in 56% of HD patients. HD patients can be expected to have reduced 25(OH) D3 levels due to their greater inactivity, reduced exposure to sunlight, and possibly lower intake of foods that are sources of natural vitamin D (fish, milk, cream and butter‏(. A low calcium diet (frequent in CKD patients) leads to a greater conversion of 25(OH)D3 to 1,25(OH)2D3, increasing the demand for vitamin D intake (7,21).

The hyperparathyroidism was observed in 30% of our patients (n**=8) that may be caused by a reduction in 1,25(OH)_2_D3 and retention of phosphorus, producing a tendency to a decrease in calcium (7,22). Metabolic syndrome increases the risk of developing many chronic diseases and originates early in life (23).

In our study, dialysis adequacy was very good (85%), however there was no significant association between adequacy of dialysis and CKD-MBD osteoporotic or osteopenic patient.

To our knowledge, this is the first study ever done for the determination of relationship between BMD and biochemical markers of bone turnover in Iranian HD children.

## Conclusion

In our study a majority of patients with CKD had low level of BMD. In addition, lower level of Ca, P, PTH, 25 (OH) vitamins D was found in patients with abnormal BMD Z-scores.

## Limitations of the study

The low number of patients is a limitation of our study. CKD is uncommon during childhood; however, we were able to recruit almost all CKD patients within our hospital region.

## Acknowledgments

We would like to thank Dr. Farahnak Assadi, the Emeritus Professor of Department of Pediatrics, Rush University Medical College, Chicago for his excellent scientific editing.

## Authors’ contribution

NH, and MM participated in all experiments, coordinated the data-analysis and contributed to the writing of the manuscript. DF coordinated the acquisition of data. MM and NS designed the research plan and organized the study. MQ performed analysis and interpretation of data. MM prepared the final manuscript.

## Conflicts of interest

The authors declare no conflict of interest.

## Ethical considerations

Ethical issues (including plagiarism, data fabrication, double publication) have been completely observed by the authors.

## Funding/Support

This article is extracted from nephrology thesis of Mehryar Mehrkash. This study was supported by a grant from Tehran University of Medical Sciences (No: 125, 2013).
